# Supplementary material for: Investigation of Vacancy-Ordered Double Perovskite Halides A2Sn1−xTixY6 (A = K, Rb, Cs; Y = Cl, Br, I): Promising Materials for Photovoltaic Applications
Source: Nanomaterials (Basel). 2023 Oct 11;13(20):2744. doi: 10.3390/nano13202744 (PMC10609051; doi:10.3390/nano13202744)
Supplement: Supplementary file 1 [file nanomaterials-13-02744-s001.zip › nanomaterials-2617925-supplementary.pdf]

Table S1. the optimized lattice parameters (Å)and volume ($Å^3$).

		X=0	X=0.25	X=0.5	X=0.75	X=1
$K_2Sn_{1-x}Ti_xCl_6$	a	10.243	10.190	10.125	10.083	10.025
	b	10.243	10.190	10.148	10.083	10.025
	c	10.243	10.190	10.148	10.083	10.025
	v	1074.6	1058.1	1042.6	1025.0	1007.4
$K_2Sn_{1-x}Ti_xBr_6$	a	10.850	10.795	10.698	10.667	10.613
	b	10.850	10.795	10.741	10.667	10.613
	c	10.850	10.795	10.741	10.667	10.613
	v	1277.5	1258.1	1234.3	1213.8	1195.5
$K_2Sn_{1-x}Ti_xI_6$	a	11.763	11.692	11.603	11.558	11.496
	b	11.763	11.692	11.637	11.558	11.496
	c	11.763	11.692	11.637	11.558	11.496
	v	1627.5	1598.4	1571.2	1544.2	1519.3
$Rb_2Sn_{1-x}Ti_xCl_6$	a	10.439	10.390	10.319	10.282	10.239
	b	10.439	10.390	10.338	10.282	10.239
	c	10.439	10.390	10.338	10.282	10.239
	v	1137.7	1121.5	1102.8	1087.0	1073.4
$Rb_2Sn_{1-x}Ti_xBr_6$	a	11.000	10.941	10.862	10.827	10.790
	b	11.000	10.941	10.887	10.827	10.790
	c	11.000	10.941	10.887	10.827	10.790
	v	1331.0	1309.7	1287.6	1269.3	1256.0
$Rb_2Sn_{1-x}Ti_xI_6$	a	11.865	11.809	11.739	11.674	11.613
	b	11.865	11.809	11.757	11.674	11.613
	c	11.865	11.809	11.757	11.674	11.613
	v	1670.1	1646.6	1622.7	1591.0	1566.2
$Cs_2Sn_{1-x}Ti_xCl_6$	a	10.757	10.704	10.661	10.623	10.576
	b	10.757	10.704	10.666	10.623	10.576
	c	10.757	10.704	10.666	10.623	10.576
	v	1244.7	1226.6	1212.8	1198.7	1182.9
$Cs_2Sn_{1-x}Ti_xBr_6$	a	11.268	11.189	11.154	11.112	11.064
	b	11.268	11.189	11.167	11.112	11.064
	c	11.268	11.189	11.167	11.112	11.064
	v	1430.5	1400.9	1391.1	1372.2	1354.5
$Cs_2Sn_{1-x}Ti_xI_6$	a	12.056	11.970	11.913	11.873	11.821
	b	12.056	11.970	11.934	11.873	11.821
	c	12.056	11.970	11.934	11.873	11.821
	v	1752.1	1714.7	1696.8	1673.9	1652.0

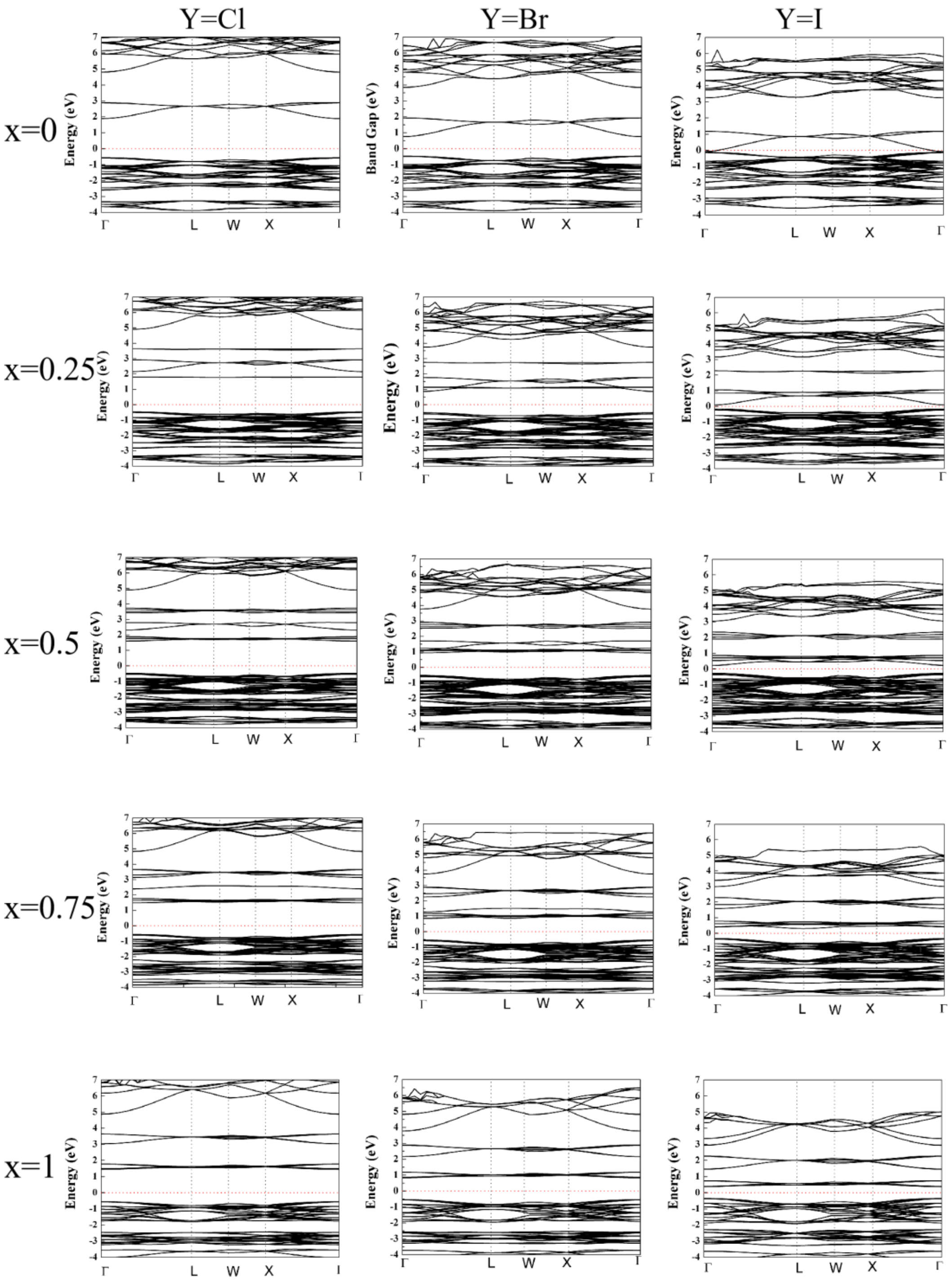

Figure S1. Band structures of $K_2Sn_{1-x}Ti_xY_6$.

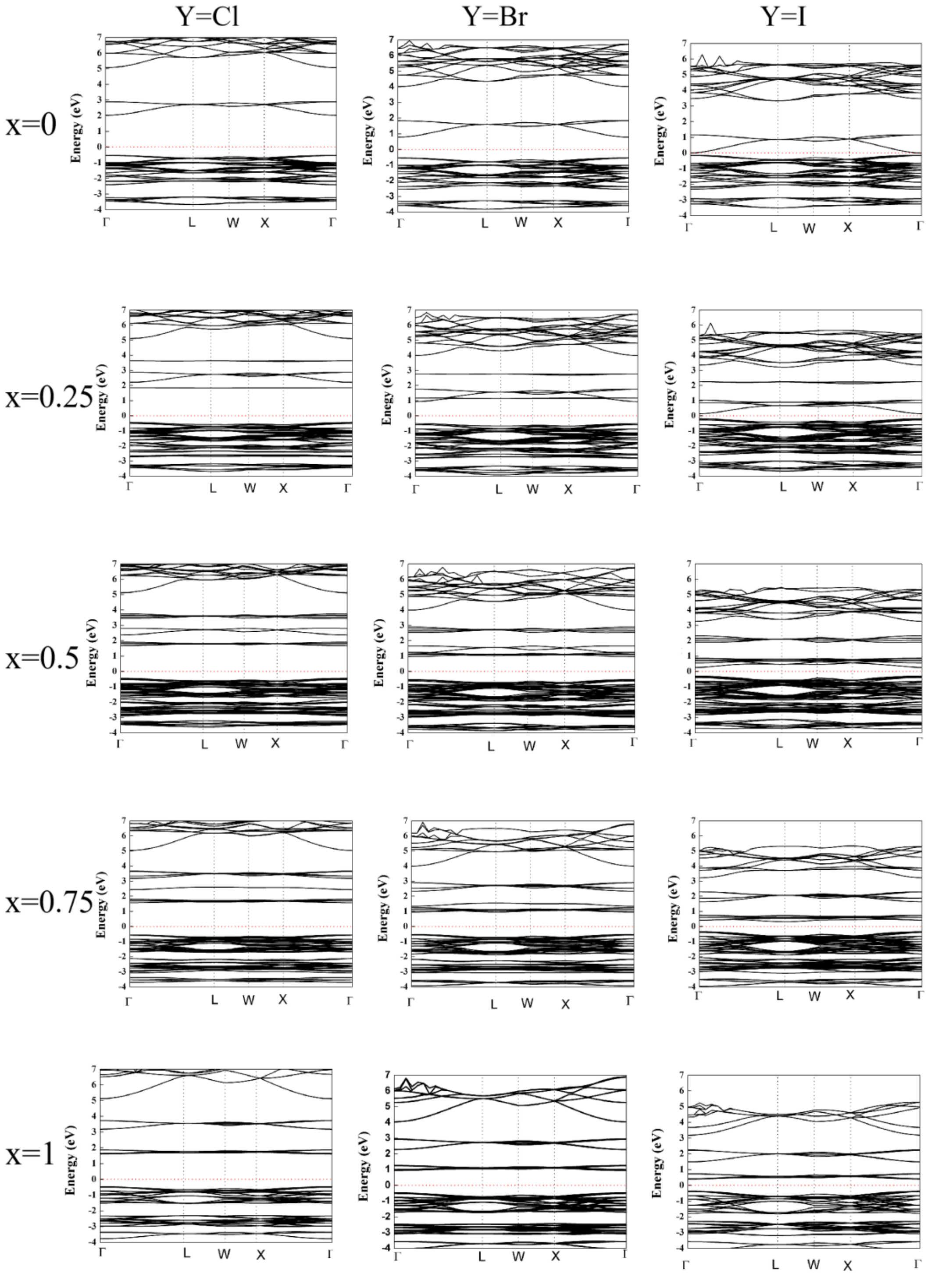

Figure S2. Band structures of $Rb_2Sn_{1-x}Ti_xY_6$.

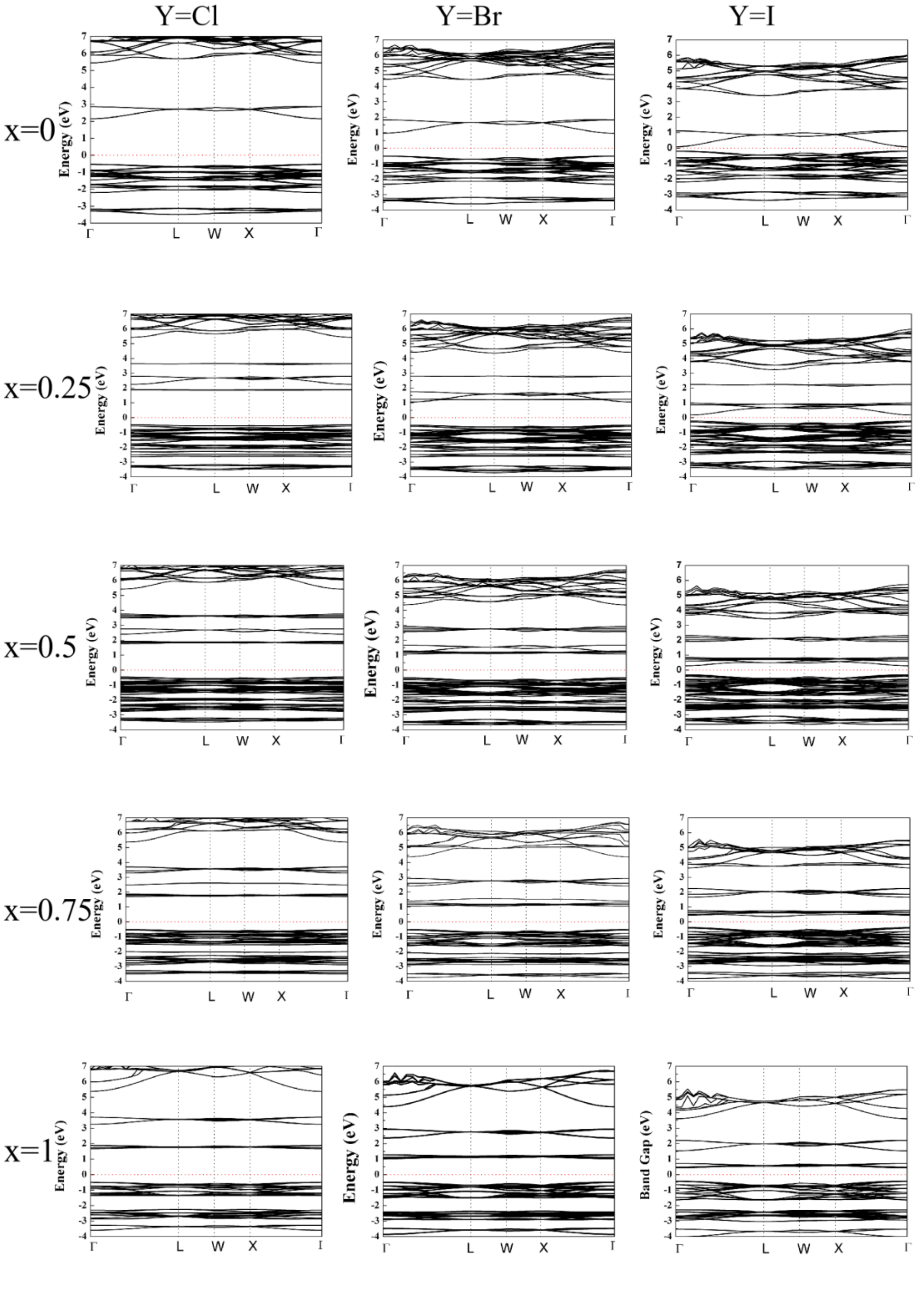

Figure S3. Band structures of $Cs_2Sn_{1-x}Ti_xY_6$.
